# Intravenously Administered Nonsteroidal Anti-Inflammatory Drugs in Clinical Practice: A Narrative Review

**DOI:** 10.3390/pharmacy13010018

**Published:** 2025-02-04

**Authors:** Axel Maurice-Szamburski, Cyril Quemeneur, Romain Rozier, Philippe Cuvillon, Claude Ecoffey

**Affiliations:** 1 Department of Anesthesiology and Critical Care, Pasteur University Hospital, 06300 Nice, France; 2 Clinique Drouot Sport, 75009 Paris, France; 3 Anesthesia and Intensive Care Department, Raymond Poincaré Hospital, APHP, 92380 Garches, France; 4 Department of Anesthesiology and Critical Care, L’Archet University Hospital, 06200 Nice, France; 5 Department of Anesthesiology, Intensive Care and Perioperative Medicine, Clinical Epidemiology, Public Health, and Innovation in Methodology, CHU Nimes, University Montpellier, 30908 Nimes, France; 6 Department d’Anesthésie Réanimation and Médecine Péri Opératoire, Hôpital Pontchaillou, Université Rennes, 35000 Rennes, France

**Keywords:** NSAID, diclofenac, ibuprofen, ketoprofen, paracetamol, acetaminophen, coxib, pain, surgery

## Abstract

Intravenously administered nonsteroidal anti-inflammatory drugs (NSAIDs) constitute a crucial component of multimodal analgesia strategies in surgical settings. This narrative review aims to provide an up-to-date evaluation of the efficacy, safety, and clinical use of intravenous (IV) NSAIDs for perioperative pain management in adults and children. The NSAIDs and selective COX-2 inhibitors (coxibs) approved in Europe for the short-term symptomatic treatment of acute, moderate perioperative pain via IV infusion in adults and/or children have been influenced by US and global guidelines and practice: the drugs primarily reviewed here are ibuprofen, ketorolac, ketoprofen, naproxen, paracetamol, and acetylsalicylic acid. Furthermore, intravenous ibuprofen is authorized for the short-term symptomatic treatment of fever. In contrast to intravenous ketoprofen, intravenous ibuprofen is authorized for administration to children over 6 years of age or weighing more than 20 kg. Overall, IV ibuprofen had a more favorable profile with regard to peri- and postoperative opioid sparing and pain relief. Oral ibuprofen and IV ibuprofen have similar levels of efficacy, although IV ibuprofen has a shorter onset of action and is required in patients who are unable to take oral medications. The frequency of significant adverse events appears to be similar for ibuprofen and paracetamol. Systematic reviews and meta-analyses report that intravenous NSAIDs reduce postoperative opioid consumption by approximately 20–60%, improving pain management with fewer opioid-related side effects. In indications in infants, the choice of medication is limited, and the oral route is not always feasible; IV formulations of ibuprofen are preferred in this setting. Topics for further research should include head-to-head trials of IV NSAIDs.

## 1. Introduction

Ensuring the sufficient relief of pain (whether acute or chronic) remains a major clinical issue [1]. Specifically, the inadequate relief of acute pain after surgery not only degrades the patient’s quality of life but may also slow wound healing, increase the risk of adverse events, accentuate the development of chronic pain, and worsen treatment observance [2,3].

Opioids are commonly utilized for acute pain relief in perioperative pain management [4] but their high level of effectiveness is accompanied by significant concerns—including the notable issues of misuse and addiction observed, particularly in the USA [5]. In pharmacological terms, centrally-acting opioids do not counter local, pain-inducing inflammation, do not have antipyretic activity, and may trigger severe adverse drug reactions, such as confusion, nausea, constipation, pruritus, urinary retention, ileus, over-sedation, and respiratory depression [6,7].

In order to prevent or reduce these drawbacks, opioids may be supplemented or replaced with other analgesic medications and therapies. In 1986, the World Health Organization introduced a framework to help non-specialist physicians manage cancer pain; this “analgesic ladder” emphasized the use of nonsteroidal anti-inflammatory drugs (NSAIDs) for mild pain, with the option to escalate to “weak” opioids for moderate pain and “strong” opioids for severe pain [8,9,10]. The concept of a bidirectional strategy (emphasizing that the intensity of pain management can also be decreased, when appropriate) was subsequently introduced [10,11,12]. However, this intensity-based WHO analgesic ladder has several shortcomings: it is overly simplistic, it does not take good account of neuropathic pain or the acute vs. chronic nature of pain, and it tends to suggest that a given step on the ladder requires a single treatment strategy. Furthermore, the concepts of “adjuvant” analgesics and “weak” vs. “strong” opioids have become outdated. Hence, the model has since evolved to reflect progress in understanding and managing pain. In 2010, Lussier and Beaulieu published a taxonomy of analgesics based on the physiological mechanism of action: antinociceptives (including paracetamol, NSAIDS, opioids and cannabinoids), antihyperalgesic drugs (including NMDA antagonists, anti-epileptics, and nefopam), compounds that modulate the descending pain pathway (including tricyclic antidepressants, and serotonin and noradrenalin reuptake inhibitors), compounds that modulate peripheral transmission and sensitization (including local anesthetics, carbamazepines, topiramate, and capsaicin), and mixed compounds [13]. Lussier and Beaulieu’s taxonomy encourages the physician to diagnose the source of pain precisely, and fits well with the multimodal analgesia concept that arose progressively in the late 1980s and early 1990s; i.e., the use of several treatment modalities that target different receptors along the pain pathway [14].

Nonsteroidal anti-inflammatory drugs (NSAIDs) have a significant role in perioperative analgesia as antinociceptive analgesics with anti-inflammatory properties. Indeed, NSAIDs have become the cornerstone of a multimodal strategy designed to decrease postoperative opioid usage, pain, and inflammation, while also reducing the risk of opioid-related adverse effects [15]. Conventional NSAIDs act by competitively inhibiting the central and/or peripheral cyclooxygenases (COXs) responsible for converting arachidonic acid into pro- and/or anti-inflammatory prostaglandins, prostacyclin, and thromboxanes; this action is thought to be responsible for the efficacy of NSAIDs (mainly when the inflammation-induced COX-2 isoform is inhibited) but also adverse drug reactions (mainly when the constitutively expressed COX-1 isoform is inhibited) [16]. Oral NSAID use has been linked to gastrointestinal track complications (such as nausea, vomiting, pain, flatulence, diarrhea, and constipation) in a duration- and dose-dependent manner, although the risk varies markedly from one drug to another and one patient population to another [17,18,19]. A multicenter case-control study in children showed that even short courses of oral treatment with an NSAID or acetylsalicylic acid were associated with an elevated adjusted odds ratio [95%CI] (vs. controls) of these complications: 3.7 [2.3–5.9] for ibuprofen, 2.6 [1.2–5.6] for ketoprofen, and 2.5 [0.9–7] for acetylsalicylic acid [20]. In a network analysis of studies of NSAID-treated patients with arthritis, naproxen was associated with a higher incidence of renal events and edema, whereas ibuprofen was associated with a higher incidence of cardiovascular events and hypertension [21]. Ketoprofen and ibuprofen inhibit COX-1 more than COX-2 but are usually considered to be non-selective COX inhibitors [22]. However, Henry et al.’s systematic review linked ketoprofen’s higher selectivity for COX-1 to greater gastrointestinal toxicity, whereas ibuprofen had the lowest risk among the studied NSAIDs [23].

The beneficial effects of COX-2 inhibition prompted the development of selective COX-2 inhibitors (coxibs) with a lower potential for serious gastrointestinal adverse effects than conventional NSAIDs [24,25,26]. However, it has been reported that even selective COX-2 inhibitors have enough COX-1-inhibiting activity to affect the gastric synthesis of prostaglandin E2 associated with adverse events [27]. Furthermore, the observation of cardiovascular adverse effects during long-term use led to the market withdrawal of rofecoxib and valdecoxib [28].

The core principle of multimodal postoperative analgesia including paracetamol and/or NSAIDs (in the absence of contraindications, such as patients undergoing coronary artery bypass graft surgery) has been endorsed as a strong recommendation, with high-quality evidence by the American Pain Society, the American Society of Regional Anesthesia and Pain Medicine, and the American Society of Anesthesiologists’ Committee on Regional Anesthesia, Executive Committee, and Administrative Council [29]. The guideline suggested that IV and oral administrations were similar for pain relief, but the onset of action might be faster with IV administration [29]. According to the guidelines published by the European Society for Paediatric Anaesthesiology (EPSA), the consistent intra- and postoperative use of non-opioid drugs (including NSAIDs) has an opioid-sparing effect and is a cornerstone of intraoperative pain management [30,31]. The EPSA recommends (if available) an intravenous paracetamol/NSAID after the induction of anesthesia and throughout the postoperative period for several types of surgery, including limb fracture repair, Nissen fundoplication (open and laparoscopic), thoracoscopy/thoracotomy, appendicectomy, hypospadias repair, and the correction of congenital hip dislocation [30,31]. The EPSA’s dose-level suggestions are 0.5 to 1 mg per kg (up to 30 mg) for a single intraoperative dose, and 0.15 to 0.2 mg per kg (up to 10 mg) every 6 h (for no more than 48 h) for ketorolac, 1 mg per kg every 8 h for ketoprofen, and 10 mg per kg every 8 h for ibuprofen [31].

In terms of pharmacokinetics (PK; see below for more details), NSAIDs are rapidly absorbed and bind strongly to plasma proteins (mainly albumin) [32,33,34,35]. Oral, rectal, and IV doses of ibuprofen tend to give similar area under the curve (AUC) values. Unsurprisingly, the peak plasma concentration (C_max_) value is higher for IV administration than for oral administration. For ibuprofen, the physiologically inactive R isomer binds even more strongly than the active S isomer. Following IV and oral administration, the respective elimination half-lives are similar. Ibuprofen is mostly oxidized by cytochromes P450 2C9 and 2C8 in the liver, with subsequent renal excretion of the metabolites [36].

The primary objective of the present narrative review is to provide an updated evaluation of the efficacy, safety, and clinical use of intravenous NSAIDs for perioperative pain management in adults and children, in order to highlight certain aspects of IV NSAID use. The secondary objective is to identify potential future trends in the use of IV NSAIDs.

We searched the MEDLINE database (via the PubMed web portal) for publications by using logical combinations of the following keywords in English: intravenous, IV, infus*, diclofenac, ibuprofen, ketoprofen, paracetamol, acetaminophen, aspirin, NSAID, nonsteroidal anti-inflammatory drug, pain, relief, surgery, non-opioid, analg*, preoperative, pain, inflamm*, perioperative, pre-emptive, postoperative, fever, combination, fixed-dose, multimodal, children, child, infant, adult, coxib, COX-1, COX-2, and cyclo-oxygenase. We focused on publications in the 10 years prior to 1 May 2024. The titles and abstracts listed in the search report were screened by one of the investigators (A M.-S.). Relevant publications in the investigators’ personal collections (including French-language publications) were also considered. Case reports, conference abstracts, oral abstracts, studies describing the results of solely in vitro studies, and publications for which a full-text version was unavailable, were excluded. The full-text versions of publications of interest were assessed by all the investigators.

## 2. Indications

### 2.1. Indications and Formulations for IV NSAIDs in Adults

The list of NSAIDs and coxibs approved in Europe for the short-term symptomatic treatment of moderate, acute pain via intravenous infusion in adults and/or children has been influenced by US and global guidelines and practice. For example, the European summaries of the product characteristics (SmPCs) for IV NSAIDs echo the American Heart Association’s 2007 scientific statement and recommend the lowest effective dose for the shortest duration needed to control symptoms [37]. The French National Agency for the Safety of Medicines and Health Products (ANSM—Agence Nationale de Sécurité du Médicament et des produits de santé) has approved ibuprofen, ketoprofen, and parecoxib solutions for infusion for the short-term treatment of postoperative pain. The French SmPCs state that intravenous administration is only justified when other routes of administration are not possible. The IV ibuprofen product for use in adults is typically supplied as a 100 mL solution containing 400 mg ibuprofen (i.e., 4 mg/mL), with infusion every 6 to 8 h as required and no more than three administrations over a given 24-h period. The recommendation infusion time is 30 min. Combination with other NSAIDs or aspirin should be avoided, and combination with the platelet aggregation inhibitor ticlopidine is prohibited. In contrast to the prescribing guidelines in some countries, treatment with lithium is not an absolute contraindication for IV NSAIDs; however, close monitoring of the blood lithium concentration is recommended. In some countries (e.g., France), the same IV ibuprofen product is also authorized in indications of fever [38,39,40].

Ketoprofen for IV use in individuals aged 15 or over is supplied as a powder (100 mg for reconstitution in 100 to 150 mL isotonic glucose solution or saline) or as a bag containing 100 mL of a 1 mg/mL solution. The recommended infusion time is 20 min, and the 24-h limit is 100 to 300 mg (i.e., one to three doses). Again, combination with coxibs or other NSAIDs should be avoided, and close monitoring of patients treated with lithium is recommended.

Parecoxib for IV use in adults is supplied as a powder (containing 40 mg of active substance) for reconstitution in 2 mL of 0.9% sodium chloride (solution). The initial dose of 40 mg can be followed every 6 to 12 h by a dose of 20 mg or 40 mg. The 24-h limit is 80 mg. Opioid analgesics can be used concurrently with parecoxib.

### 2.2. Indications and Formulations for IV NSAIDs in Children

Children often have variable renal function, protein binding, and metabolic pathways, which can alter NSAID pharmacokinetics. Current labeling restricts IV formulations of some NSAIDs to older children, leaving limited approved options for infants and toddlers. Clinicians must therefore apply weight-based dosing and monitor closely for adverse events. Ongoing research is needed to refine age-specific guidelines and extend licensing to broader pediatric populations.

In contrast to the USA (where the indications for IV ibuprofen were extended to pediatric patients as young as 6 months in 2015), the lower limit for the administration of IV ibuprofen in France is 6 years of age or a bodyweight of 20 kg. In that case, the drug is supplied as a 50 mL solution containing 200 mg of ibuprofen (i.e., 4 mg/mL, the concentration used in adults). The recommended dose is 20 to 30 mg/kg of body weight administered in three to four doses (5–10 mg/kg), with a 24-h maximum cumulative amount of 600, 800, or 1200 mg, depending on bodyweight. In contrast to IV ibuprofen, ketoprofen solution for infusion has not been approved for administration to patients under the age of 15. Nevertheless, a report from the French health authorities in 2022 acknowledged that off-label use of ketoprofen solution for infusion in children between 1 and 15 years of age (i.e., including those below 6 years of age, the current age cut-off) was widespread [39]. This off-label use by practitioners might be encouraged by clinical trial data showing that the pharmacokinetic and short-term safety profiles of IV ibuprofen in very young patients (1–6 months of age) are similar to those in older children [41]. Lastly, diclofenac solutions for infusion (25 mg/mL) are authorized for short-term use in patients aged 16 and over in the following indications: acute rheumatic inflammation, acute back pain, nerve root pain, and renal colic; hence, diclofenac for infusion is not indicated in general surgical settings.

## 3. Studies of the Efficacy, Safety, and Pharmacokinetics of IV NSAIDs for the Relief of Acute Perioperative Pain

### 3.1. Studies in Adults

The wide perioperative use of intravenously administered NSAIDs has prompted extensive debate and research on whether one of these drugs is safer and/or more effective than the others [42]. The relative lack of randomized, head-to-head comparative trials of IV NSAIDs makes it hard to answer this question; most trials have been placebo-controlled, rather than active-comparator-controlled (Table 1) [43,44,45,46,47,48,49,50,51,52,53,54,55,56,57,58,59,60,61,62,63,64,65,66,67,68]. In one head-to-head trial, Kostamovaara et al. compared treatment with IV ketorolac, IV diclofenac, or IV ketoprofen for pain relief after total hip replacement surgery in 85 patients; there were no significant differences between these three NSAIDS with regard to pain scores or opioid consumption [69].

A network meta-analysis extensively reviewed the benefits of NSAIDs vs. other non-opioid analgesics, highlighting their ability to significantly reduce opioid use and reduce pain, especially when combined with drugs such as paracetamol or nefopam. The analysis calls for more direct comparisons and improved reporting on serious adverse events to better assess the balance between efficacy and safety [42].

With regard to safety, two meta-analyses published in 2011 and 2012 found that ibuprofen (administered intravenously or orally) had a more favorable profile than diclofenac and ketorolac with regard to upper GI and cardiovascular adverse events [70,71]. Koh et al.’s 2015 review of a small number of randomized clinical trials (RCTs) of NSAIDs for peri- and postoperative pain management found that intravenously administered ibuprofen was associated with significant opioid sparing and was well tolerated.

In 2020, Southworth et al. updated their previous safety analysis by publishing a narrative summary of the results of nine clinical studies of perioperative pain management with IV ibuprofen. The studies covered 1062 adults, 757 of whom received IV ibuprofen and 305 of whom received either a placebo or a comparator drug [62,72]. The researchers concluded that the administration of IV ibuprofen was associated with lower postoperative pain levels, lower opioid use, better recovery, less fatigue, a less intense surgical stress response (i.e., lower levels of catecholamines, cortisol, and cytokines), and less postoperative use of over-the-counter medication [72]. Zhou et al.’s meta-analysis (published in 2023) considered 32 studies (with 3716 participants and 18 types of surgery) of multiple-dose or single-dose IV ibuprofen [40]. The level of evidence was judged to be low-moderate. With regard to postoperative pain, IV ibuprofen gave lower scores than placebo (mean difference: −3.53 at 0 min and −0.96 at 24 h) and IV paracetamol (mean difference, −1.54 at 0 min and −0.36 at 24 h). In terms of antipyretic activity, IV ibuprofen and IV paracetamol showed similar, satisfactory levels of effectiveness. The researchers concluded that their results supported the use of IV ibuprofen for adults with postoperative pain and fever, and those who are unable to take oral medications [40].

These results are in line with the large body of safety data for orally administered ibuprofen and paracetamol in a setting of acute pain. For example, Moore et al.’s PAIN study of 8677 patients with acute pain found that the frequency of significant adverse events was similarly and significantly lower for ibuprofen and paracetamol (13.7% and 14.5%, respectively) than for aspirin (18.7%) [73,74]. Lastly, the market withdrawals of the coxibs rofecoxib and valdecoxib focused attention on the safety of NSAIDS more generally and prompted Thomas et al. to conclude that the “preferred NSAIDs are ibuprofen and naproxen” [75].

### 3.2. Studies in Children

In a recent Cochrane Collaboration review of diclofenac for the relief of acute postoperative pain in children (covering 32 RCTs and 2250 children, most of whom were over the age of three), Ringsten et al. emphasized their uncertainty about the efficacy of this NSAID over placebo and active comparators, regardless of the administration route [76]. However, the researchers pointed out that this uncertainly was mainly due to the absence of data on clinically important outcomes in some trials and/or poor trial conduct. In a separate Cochrane Collaboration review, the same group of researchers found that this uncertainly also applied to studies of the relief of acute postoperative pain in children by ibuprofen [77]. Nevertheless, on the basis of three studies in 259 children, Pessano et al. concluded that there was moderate-certainty evidence to suggest that ibuprofen reduces child-reported pain intensity less than 2 h post-surgery, relative to placebo (standard median difference [95%CI] = −1.12 [−1.39 to −0.86]) and low-certainty evidence for an effect up to 24 h post-surgery [77]. According to Pessano et al., the low-certainty evidence was largely due to poor reporting on serious adverse events, poor study conduct, or poor reporting.

In the studies reviewed here, the overall safety profile appeared to be similar for ibuprofen, paracetamol, and ketorolac (Table 1). Perioperative adverse events (especially bleeding) were often infrequent or absent [46,54,59,63,64,66]. In Ahiskalioglu et al.’s study, the incidences of nausea and vomiting were lower even in the ibuprofen group than in the saline placebo group [43].

Overall, PK data for IV NSAIDs in infants are scarce. According to Khalil et al.’s multicenter study of 43 pediatric inpatients receiving IV ibuprofen, the area under the curve (AUC) from time zero to 4 h (AUC 0–4) ranged from 22.96 to 162.06 µg.h/mL, and the peak plasma concentration (C_max_) was usually around 60 µg/mL and ranged from 15.91 to 96.31 µg/mL [78]. The median time to C_max_ (t_max_) was 10 min, and the mean (range) elimination half-life was 1.55 h (0.79–2.87). As expected, the clearance and distribution volume increased with age. By way of a comparison, the value of tmax for single-dose oral administration achieving the same Cmax is reportedly 1.5 to 2 h [79]. Kokki et al. reported a mean (range) elimination half-life of 1.3 (0.8–1.7) h for a 24-continuous infusion and 1.5 (0.7–3.0) h for a single intravenous injection of ketoprofen 1 mg/kg [80,81]. The steady-state plasma concentration of ketoprofen was 2.0 µg/mL (range: 1.3–2.7) for a 24-continuous infusion, and C_max_ ranged from 10.5 to 22.2 µg/mL for a single injection (tmax was not reported) [80,81]. For single IV doses (0.5 or 1 mg/kg) of IV ketorolac in infants, Lynn et al. reported respective mean ± SD C_max_ values of 1.1 ± 0.6 and 2.0 ± 11 for S-ketorolac and 2.5 ± 1.4 and 4.1 ± 1.8 for R-ketorolac [82].

### 3.3. Comparisons of NSAIDs with Paracetamol

Intravenous formulations of paracetamol were approved in Europe in 2002 and in the USA in 2009 for the treatment of mild-to-moderate pain and (when combined with opioids), severe pain. The main safety concern with paracetamol is hepatotoxicity if used above the recommended maximum dose of 4 g per day in adults. Although often used as an active comparator in trials of NSAIDs such as ibuprofen and ketorolac, paracetamol has very weak peripheral anti-inflammatory effects and lacks an effect on platelet function [83]. Koh et al.’s 2015 review of studies of peri- and postoperative pain management found that like ibuprofen, IV paracetamol was associated with significant opioid sparing and was well tolerated [84].

Qureshi et al. systematically reviewed the literature (27 trials; 5427 patients) on the use of intravenous paracetamol, intravenous or intramuscular NSAIDs, and intravenous opioids for the relief of moderate-to-severe pain in the emergency department. The researchers found that all three drug classes provided similar levels of pain relief but that opioids were associated with more adverse events and intravenous paracetamol was associated with a greater need for rescue analgesia. Hence, Qureshi et al. recommended NSAIDs as the first-choice analgesia [85]. With regard to efficacy, Dogan et al.’s recent randomized, double-blind study of 210 trauma-free patients with acute low back pain did not evidence a significant difference in pain relief (0-, 15-, 30- and 60-min post-infusion) between IV paracetamol, IV dexketoprofen, and IV ibuprofen [86].

### 3.4. Combining an NSAID with Paracetamol

According to Crook, the UK National Institute for Health and Clinical Excellence recommended that ibuprofen and paracetamol should be used separately because there was no evidence of greater effectiveness when combined [38]. Somewhat in contrast, Hamdi et al. considered that the continuous infusion of ketoprofen in a binary mixture with paracetamol, nefopam, or ketamine was safe [87]. Martinez et al. performed a network meta-analysis of 135 randomized trials (13,287 patients) of non-opioid analgesics in adults after major surgery, looking at morphine consumption, pain, and adverse effects. The researchers concluded that with regard to the reduction of opioid consumption, a combination of paracetamol with an NSAID or nefopam was superior to most non-opioid analgesics used alone. NSAIDs and COX-2 inhibitors alone were superior to paracetamol alone [42]. For example, Gupta et al.’s RCT in the USA found that perioperative IV ibuprofen combined with IV paracetamol was associated with significantly greater pain relief (but on POD 3 only), lower opioid use, and fewer opioid-related adverse events when compared with IV ibuprofen alone [50]. In contrast, in the context of total hip arthroplasty and oral administration, Thybo et al. found that the combination of ibuprofen and paracetamol did not result in a clinically significant improvement over ibuprofen alone [88]. Furthermore, the guideline on postoperative pain management after total hip arthroplasty issued by the PROSPECT Working Group and the European Society of Regional Anaesthesia and Pain Therapy states that IV paracetamol has a limited impact when added to a regimen including coxibs or NSAIDs [89].

## 4. Study Strengths and Limitations

The present narrative review has several strengths. Firstly, we covered a broad, topical issue with immediate clinical relevance. Secondly, we reviewed publications available solely in French, as well as those in English.

Our review also has limitations. Firstly, it was not systematic. However, several systematic reviews of some of the subtopics addressed here are available [40]. Secondly, in view of the investigators’ affiliation, the review was centered on practice in France and Europe, and cannot be extended to all healthcare systems and clinical frameworks. Thirdly, five of the studies reviewed here in detail were published after 2020 and were conducted during or following the global pandemic of coronavirus disease 2019 (COVID-19). Early in the pandemic, unsubstantiated media reports suggested that NSAIDs might exacerbate the signs and symptoms of COVID-19. The results of a number of robust clinical studies have now demonstrated that NSAID use is not associated with worse COVID-19 outcomes, and the European Medicines Agency (EMA), and the US Food and Drug Administration (FDA) did not advise against using NSAIDs [90,91,92,93]. On the contrary, some studies even found a modest survival benefit for treatment with NSAIDs [91].

A shortage of head-to-head RCTs comparing different IV NSAIDs limits direct comparisons. Most data come from placebo-controlled or observational studies, which can restrict the accuracy of clinical decision-making. Future trials should assess the comparative efficacy, safety, and cost-effectiveness of various IV NSAIDs in diverse populations.

## 5. Conclusions

Our review of the literature data in various surgical settings indicated that intravenous NSAIDs are well tolerated and usually associated with significant (~20–60%) opioid sparing, postoperative pain relief, and fewer side effects. Intravenous formulations of NSAIDs will probably continue to be of value in the multimodal approach to perioperative analgesia and enhanced recovery after surgery. In pediatrics in particular, the choice of medication is limited to ibuprofen at early ages and the oral route is not always feasible; hence, IV formulations of NSAIDs are the preferred allies in this setting. Topics for further research should include head-to-head trials of IV NSAIDs. In this respect, the American Pain Society’s Clinical Practice Guideline on acute postoperative pain recommends (i) both RCTs and observational studies that capture real-world data and rare AEs, (ii) the stratification of groups according to the baseline pain level, (iii) standardized outcome measures for pain, function, safety, harms, and patient satisfaction, and (iv) measurement periods extending over days and weeks, rather than hours [94].

## 6. Future Directions

### 6.1. Continuous Infusion vs. Intermittent Infusion

One can hypothesize that continuously infused NSAIDs are superior to intermittent dosing due to the pre-emptive inhibition of inflammation and thus greater pain relief. Continuous infusion also limits the peak concentration of the drug and so might have safety benefits. Indeed, multiple intermittent doses of IV ibuprofen led to a slight accumulation of the drug in some studies [15] but not in others [95]. On the one hand, continuous infusion reduces the number of handling procedures by the nursing staff. On the other, the infused NSAIDs must be compatible with other infused products.

Howard et al. have extensively reviewed studies of continuous NSAID infusion lasting more than 12 h against intermittent dosing in various surgical settings [4]. In the literature on abdominal surgery, ketorolac was the most commonly reported continuously infused NSAID; of the 10 trials of ketorolac, half did not find a difference in reported pain levels at any of the time points, and half found a difference in reported pain levels at some time points [4]. The benefits in terms of reduced morphine consumption were more visible, especially when patient-controlled analgesia was available. Howard et al.’s analysis was limited by heterogeneity in study outcomes, the lack of head-to-head comparisons between continuous infusion and intermittent dosing of the same NSAID, and a relatively short observation period per patient (which limited the detection of medium- and long-term adverse events). The researchers concluded that in orthopedic surgery for adults, there was strong evidence of benefits in pain relief and opioid sparing for continuously infusion NSAIDs vs. placebo, especially at doses approaching the maximum recommended daily level [4]. For example, the results of Ready et al.’s multicenter, double-blind, placebo-controlled study showed that at similar cumulative doses, morphine patient-controlled analgesia (PCA) use was 25% lower in the continuously infused ketorolac group than in the bolus group [96]. Unfortunately, Howard et al.’s analysis of studies in children was restricted by the scarcity of data and the lack of availability of IV ketoprofen in some countries (including the USA, notably) [4].

### 6.2. Rapid Infusion vs. Standard Infusion

The results of a Phase IV multicenter clinical surveillance study conducted in the USA and described by Bergese et al. and Gan et al. showed that rapid IV administration of ibuprofen (over 5 to 10 min, rather than the standard 30 min stated in the SmPC) was well tolerated in hospitalized patients in general and surgical patients in particular [97,98]. The most common adverse event (in 34 (11%) of the 300 surgical patients) was infusion site pain, and none of the serious adverse events were judged to be related to ibuprofen.

### 6.3. Pre-Emptive Analgesia

It has been suggested that in the broad setting of multimodal analgesia, pre-emptive analgesia can reduce pain levels and opioid use in the days following surgery, notably for lower third molar removal, tonsillectomy, total knee or hip arthroplasty, hysterectomy, and lumbar spine surgery [99,100,101,102]. Pre-emptive analgesia with IV formulations of NSAIDs has been evaluated in a small number of trials. For example, a series of placebo-controlled studies by Ahiskalioglu et al. found that a pre-emptive single dose of IV ibuprofen before laparoscopic cholecystectomy and other operations was associated with lower postoperative pain levels and 45% less opioid consumption in the first 24 h after surgery [43,103,104]. However, the literature data are contradictory: a recent review of five trials in lower third molar surgery found that there was insufficient evidence in favor of pre-emptive ibuprofen administration for the reduction of postoperative pain in this indication [105]. A similar conclusion was reported in a review of intramuscular or intravenous administration of ketorolac in lower third molar surgery [106]. Lastly, a recent (2022) network meta-analysis highlights NSAIDs (along with paracetamol and epidural anesthesia) as among the most effective treatments in pre-emptive analgesia [107].

### 6.4. Dosing Adjustments for Specific Patient Profiles

Given that NSAIDs are hydrophilic molecules, we suggest that the ideal body weight or lean body weight (rather than the actual body weight) should be taken into account for obese patients, as with antibiotics [108,109]. This question has also been considered for paracetamol [110].

### 6.5. Fixed-Dose Combinations

Some experts recommend fixed-dose combinations (FDCs) over the parallel administration of separate drugs for effectiveness and safety reasons [111]. In a randomized, open-label, five-period cross-over pharmacokinetic study, Atkinson et al. compared a single dose of an intravenously administered FDC of 3 mg/mL ibuprofen and 10 mg/mL paracetamol with single doses of the individual components (IV 3 mg/mL ibuprofen alone; IV 10 mg/mL paracetamol alone) and an orally administered combination (ibuprofen 150 mg + paracetamol 500 mg). With regard to the C_max_, area under curve, and bioavailability, the IV FDC did not alter the pharmacokinetic profile of either drug [112].

### 6.6. Pharmacogenomic Profiling

Pharmacogenomic questions are increasingly being raised in the field of pain and NSAID use [113]. Given their extremely wide use, NSAIDs are the primary cause of drug hypersensitivity reactions [114]. These reactions may be broad (i.e., cross-reactive) or narrow (i.e., selective) and are related to the expression levels and methylation status of immune-response genes [114]. For example, polymorphisms in the genes coding for COX-1 and COX-2 (*PTGS1* and *PTGS2*, respectively) are linked to cross-reactive NSAID hypersensitivity, and the N-acetyltransferase 2 *5, *6, *7, and *14 genotypes are associated with selective NSAID hypersensitivity [115,116]. It has been known for several decades that the metabolism of NSAIDs (including ibuprofen, celecoxib, piroxicam, and diclofenac) is influenced by cytochrome P450 polymorphisms [117]. The pharmacokinetics of S-ibuprofen and R-ibuprofen are affected by CYP2C9*2 and CYP2C9*3 polymorphisms and sex [118]. These concerns might open up long-term perspectives for the greater safety and effectiveness of IV NSAIDs.

In the era of personalized medicine, further research on inter-individual differences in reactions to and the effectiveness of IV NSAIDs is necessary.

### 6.7. Environmental Concerns

Regulatory measures are increasingly being taken to reduce the amount of plastic waste (notably microplastics and nanoplastics) and xenobiotics being generated and released into the environment, and the medical device and drug industries are not exempt from such considerations [119,120,121]. Due to the resources consumed and the large amount of waste produced, operating theaters contribute significantly to a hospital’s environmental impact [122]. The production and supply of ready-to-use products (e.g., prefilled syringes of commonly used anesthetic drugs) might reduce production waste, favor the use of lighter and more recyclable inner and outer packaging, and reduce phthalate and bisphenol A contents [123]. It has been reported that IV paracetamol has an environmental impact that is 8 to 16 times greater than that of the orally administered drug [124]. We suggest that ready-to-use IV formulations may be preferred by some users and may be well suited (but not limited) to certain patient profiles (e.g., infants, with a lower likelihood of dosing errors) and clinical settings (e.g., emergency departments, where rapid preparation is an advantage).

It remains to be seen whether these environmental factors are considered by physicians and administrators when choosing between IV and oral NSAID formulations, or between a ready-to-use IV formulation and an IV formulation requiring preparation.

## Figures and Tables

**Table 1:** Clinical studies of perioperative IV infusion of ibuprofen and comparators in surgery patients.

Type of Surgery (in Alphabetical Order)	Reference	Study Design	Patient Age	Number of Patients	Ibuprofen Dosing Regimen	Other Drugs	Control or Comparator	Main Results	Adverse Events
Bunionectomy	Daniels et al. 2019 [45]	DBPC RCT, 3 arms, 2 centers	18 to 65	276	Postop: IV FDC (ibuprofen 300 mg + paracetamol 1000 mg) every 6 h for 48 h	Rescue medication: primary, oral oxycodone 5–10 mg; secondary, IV morphine sulfate 2–4 mg)	Ibuprofen 300 mg or paracetamol 1000 mg, or placebo	The mean (standard error) SPID48 score was higher for the FDC (23.4 (2.5) mm) than for ibuprofen alone (9.5 (2.5) mm) or paracetamol (10.4 (2.5) mm); all *p* < 0.001 vs. placebo (−1.3 (3.1)). The proportion of patients using opioid usage was lower for the FDC (75%, vs. 92% for ibuprofen, 93% for paracetamol, and 96% for placebo).	The safety profile of the FDC was similar to that of IV ibuprofen or paracetamol alone
Cervical cancer surgery	Liu et al. 2018 [56]	Prospective, DBPC RCT, 3 arms, single center	18 to 70	60	IV ibuprofen 400 mg, or ibuprofen 800 mg 30 min before end of surgery then every 6 h for a total of 8 doses in the first 48 h	Morphine PCA, followed by tramadol	Placebo group: saline?	IV ibuprofen 800 mg was associated with a significant reduction in the morphine requirement during the first 24 h (17.6 ± 3.2 mg vs. 19.7 ± 3.0 mg with the placebo; *p* = 0.04)	Safety assessments and adverse effects were similar in the three groups
Cleft palate repair	Peng et al. 2021 [59]	Prospective, DBPC RCT, 2 arms, single center	9 to 24 months	40	Pre-emptive IV administration ibuprofen 10 mg/kg at induction	Rescue with titrating IV fentanyl 0.5 μg/kg	Saline	Significant opioid-sparing effect in early postoperative period in the ibuprofen group (mean ± SD total fentanyl dose = 3.20 ± 4.49 µg, vs. 9.40 ± 4.49 µg in the placebo group; *p* < 0.001)	No obvious adverse events reported
Inguinal and umbilical hernia repairs	Sparber et al. 2017 [63]	Prospective, DBPC RCT, single center, 2 arms	18 and older	48	800 mg of IV ibuprofen or placebo preoperatively (30 min before)	IV hydromorphone, or oral oxycodone/paracetamol, paracetamol only, or ibuprofen, or in combination	Placebo: normal saline	Pre-op IV ibuprofen did not significantly reduce postop pain	No major serious adverse events were reported
Knee arthroscopic surgery	Uribe et al. 2018 [65]	DB pilot RCT, single center, 2 arms,	18 and older	51	Ibuprofen IV, two 800 mg doses (the first 2 h before surgery)	IV hydromorphone 0.5 mg as needed	IV ketorolac: a single 30 mg dose after surgery	The use of pre-emptive IV ibuprofen 800 mg could be considered to reduce postop pain and opioid consumption. The mean ± SD time to first rescue medication was 77.62 ± 33.03 min in the ibuprofen group and 55.78 ± 35.37 in the ketorolac group (*p* = 0.0456).	No significant intergroup differences in patient satisfaction and documented AEs during the first 24 h
Knee or hip arthroplasty	Gupta et al. 2016 [50]	RCT, single center, 2 arms	26 to 70	78	Ibuprofen IV + paracetamol IV up to 5 days (peri- and postoperative)	Morphine and/or hydromorphone	Ibuprofen alone	Patients on ibuprofen + paracetamol had lower VAS scores vs. ibuprofen alone on Day 3, only 6.7 vs. 4.9, respectively (*p* < 0.002), together with lower median (range) opioid requirements (20 (5–25) vs. 25 (10–35), respectively; (*p* < 0.001))	Patients treated with ibuprofen + paracetamol experienced fewer opioid-related adverse events than those treated with ibuprofen (*p* < 0.001)
Laparoscopic cholecystectomy	Mohammadian Erdi et al. 2022 [57]	DBPC RCT, single center, 3 arms	20 to 60	90	800 mg IV ibuprofen or 1 g IV paracetamol 3 times (during the operation and 8 and 16 h after)	Fentanyl (15 μg/mL) as PCA, meperidine	Placebo: normal saline	The mean abdominal pain scores were not significantly different in the ibuprofen (3.02) and paracetamol (2.89) groups (*p* = 0.719), but both were significantly lower than in the control group (5.10; *p* < 0.001)	Shoulder pain, nausea, and vomiting were not significantly different in the ibuprofen and paracetamol groups
Laparoscopic cholecystectomy	Lee et al. 2022 [54]	Retrospective study, single center, 2 arms	18 and over	163	Pre-op IV ibuprofen (400 mg)	Tramadol 50 mg	IV ketorolac (30 mg)	Postop pain score measured in the recovery room was significantly higher in the ibuprofen group than in the ketorolac group (mean value: 5.09 vs. 4.61; *p* = 0.027)	No bleeding event of the operative site or gastrointestinal mucosa in either group
Laparoscopic cholecystectomy	Ekinci et al. 2020 [48]	DBPC RCT, single center, 3 arms	18 to 70	90	Postop: 800 mg of IV ibuprofen, or 1000 mg of IV paracetamol	Rescue opioid	Placebo: saline	Compared with postop paracetamol, IV ibuprofen resulted in a lower mean ± SD pain score (at 24 h: 1.16 ± 0.79 vs. 0.26 ± 0.44, respectively; *p* < 0.001) and reduced opioid use (342.33 ± 65.59 vs. 215.66 ± 97.26, respectively) in the first 24 h	The incidence of nausea was significantly lower (*p* < 0.05) in the ibuprofen group than in the paracetamol group
Laparoscopic cholecystectomy	Ahiskalioglu et al. 2017 [43]	Prospective, DBPC RCT, single center, 2 arms	18 to 65	60	400 mg IV ibuprofen in 100 mL saline 30 min before surgery	Postop: 1000 mg paracetamol/6 h + patient-controlled IV fentanyl	Placebo: 100 mL saline solution	24-h opioid consumption was significantly lower in the ibuprofen group (303.33 ± 132.08 mcq vs. 553.00 ± 257.04 for the placebo; *p* < 0.001). Rescue medication (meperidine) use was lower in the ibuprofen group than in the placebo group (125 mg vs. 350 mg, respectively; *p* = 0.012).	The incidences of nausea and vomiting were lower in the ibuprofen group
Laparoscopic cholecystectomy	Le et al. 2016 [53]	Prospective, DBPC RCT, 3 centers, 2 arms	18 or older	55	IV ibuprofen 800 mg preoperatively	Rescue opioids	Placebo: saline	Pre-op IV ibuprofen was associated with lower mean ± SD levels of cortisol (173.6 ± 29.5 pg/mL vs. 289.5 ± 25.9 for placebo, *p* = 0.001), lower mean ± SD intraoperative norepinephrine levels (0.52 ± 0.09 pg/mL, vs. 1.06 ± 0.12 pg/mL for placebo, *p* = 0.004), and no decline in the QoR40 recovery score	Three cases of postop nausea in the placebo group
Laparoscopic hernia repair	Lee et al. 2021 [55]	Prospective, DB RCT, single center, 3 arms	6 months to 6 years	159	10 mg/kg IV ibuprofen or 10 mg/kg IV ibuprofen + 30 mg/kg IV propacetamol during anesthesia	1.0 μg/kg fentanyl was administered as a rescue analgesic	30 mg/kg IV propacetamol	Ibuprofen + propacetamol immediately following surgery in children was associated with a lower proportion of patients using fentanyl (12.8%, vs. 28.6% for ibuprofen alone and 66.7% for propacetamol alone; *p* < 0.001)	There were no safety differences between the groups and no perioperative adverse events
Orthognathic surgery	Tomic et al. 2022 [64]	Prospective, DB RCT, single center, 2 arms	18 to 61	109	Postoperative IV 600 mg ibuprofen	Metamizole 500 mg. Rescue pain medication: paracetamol 1000 mg and piritramide 7.5 mg.	IV 75 mg diclofenac + 30 mg orphenadrine given twice daily	Ibuprofen administration was associated with less pain on the third postop day for patients who underwent bimaxillary osteotomy (1.23, vs. 2.73 for diclofenac + orphenadrine; *p* = 0.015)	No major postoperative complications or AEs due to pain medication
Orthopedic trauma surgery (fracture of the ribs, face, extremities, and/or pelvis)	Weisz et al. 2020 [67]	Prospective, DBPC RCT, single center, 2 arms	18 to 75	99	800 mg IV ibuprofen or placebo administered every 6 h for a total of 8 doses within 48 h of admission	PRN pain medications	Placebo: saline	IV ibuprofen was associated with significantly less opioid consumption compared with placebo (difference in least-square means [95%CI] = 222.9 [241.4–24.2] mg; *p* = 0.017) and greater pain reduction 8 h after start of infusion (difference in least-square means [95%CI] = 1.1 [0.2–2.0]; *p* = 0.013)	Not reported
Orthopedic surgery (knee or hip replacement, reconstruction or arthroplasty)	Singla et al. 2010 [61]	DBPC RCT, 8 centers, 2 arms	18 to 80	185	800 mg IV ibuprofen or placebo every 6 h, first dose administered preoperatively	IV morphine for rescue	Placebo: saline	Patients receiving IV ibuprofen used 30.9% less morphine (mean ± SD: 41.1 (27.3), vs. 59.5 ± 29.9 in the placebo group; *p* < 0.001) and experienced less pain (43.2 ± 3.6, vs. 51.8 ± 3.7 in the placebo group: *p* < 0.001)	Similar treatment-emergent AEs occurred in both groups
Third molar surgery	Demirbas et al. 2019 [46]	Prospective, DBPC RCT, single center, 3 arms	18 to 50	75	IV ibuprofen 60 min before surgery + IV placebo (saline) after surgery, or IV placebo before + IV ibuprofen after surgery	Rescue paracetamol	Placebo: IV saline before and after surgery	Pre-emptive use of IV ibuprofen resulted in less pain (42.6% lower) and less rescue analgesia (640 mg of paracetamol, vs. 1840 mg for placebo; *p* < 0.001) during the first 24 h	There were no postoperative complications and no adverse events in any of the groups
Third molar surgery	Küpeli and Gülnahar 2019 [52]	DBPC RCT, single center, 3 arms	20 to 35	60	Preoperative ibuprofen 800 mg IV + dexketoprofen 50 mg, or pre-op ibuprofen 800 mg IV alone	Postoperative infusion of dexketoprofen + methylprednisolone 40 mg + sultamicillin tosilate	Placebo: saline	No significant difference in postop pain scores between the two treated groups but both were lower than in the placebo group	Not reported
Third molar surgery	Viswanath et al. 2019 [66]	Single center randomized SB study, 2 arms	Median [interquartile range] = 22 [6] in the ibuprofen group	41	Preoperative IV ibuprofen (800 mg)	Postoperative analgesic (narcotic and over-the-counter)	Pre-op IV paracetamol (1000 mg)	The pain level was significantly lower in the ibuprofen group than in the paracetamol group (*p* = 0.004) at 4 h, 24 h (*p* = 0.019), and 48 h (*p* = 0.017). Ibuprofen was associated with less opioid use (2.68 ± 2.26, vs. 7.32± 6.68 in the placebo group; *p* = 0.005).	No adverse effects were reported in either group
Tonsillectomy	Cui et al. 2022 [44]	DBPC RCT, single center, 2 arms	6 months to 12 years	95	15 min before surgery with IV ibuprofen 10 mg/kg	Postop: IV fentanyl (0.5 µg /kg) when needed	Placebo: saline	Rescue fentanyl was 18% lower in the IV ibuprofen group (*p* = 0.043). There was no significant difference in the amount of fentanyl administered postop (*p* = 0.127).	No significant differences in terms of operative blood loss (*p* = 0.978), vomiting, or postop bleeding (*p* = 0.474)
Tonsillectomy	Gao et al. 2022 [49]	DBPC RCT, single center, 2 arms	3 to 9	89	During operation, dose of 10 mg/kg of IV ibuprofen slowly infused over 15 min	Fentanyl 0.5 μg/kg (max dose of 2 μg/kg) administered postop if necessary	Placebo: saline	Ibuprofen decreased the incidence of emergence agitation at 15 min after extubation (8.9%, vs. 34.1% in the control group; *p* = 0.004) and the median [interquartile range] pain score at 15 and 30 min (e.g., 1.0 (0–3.0) for ibuprofen and 3.0 (1.0–6.0) for placebo at 15 min, respectively; *p* = 0.007)	No postoperative hemorrhagic complications
Tonsillectomy	Moss et al. 2014 [58]	DBPC RCT, multicenter, 2 arms	6 to 17	161	Preoperative IV ibuprofen dose of 10 mg/kg	IV fentanyl (0.5 µg/kg) as needed	Placebo: saline	The proportion of patients requiring fentanyl was significantly lower in the IV ibuprofen group than in the placebo group (42% vs. 62%, respectively; *p* = 0.021). There was no significant difference in the time to first analgesia request.	No significant differences in the incidence of serious AEs, surgical blood loss (*p* = 0.662), or postop bleeding
Total hip replacement	Gürkan et al. 2019 [51]	Prospective, DBPC RCT, single center, 2 arms	18 to 70	40	800 mg ibuprofen IV every 6 h for 24 h, first dose 30 min before the end of surgery	After surgery tramadol 100 mg IV and paracetamol 1 g IV + morphine PCA	Not reported	In the ibuprofen group, the mean ± SD postop 24 h pain VAS was significantly lower (1 ± 1.05, vs. 2 ± 2.25 for placebo; *p* = 0.006), as was morphine consumption (14.90 ± 9.33 vs. 21.93 ± 11.35 for placebo; *p* = 0.026)	Vomiting: 5 patients in the control group and 3 patients in the ibuprofen group
Transsphenoidal surgery	Shepherd et al. 2018 [60]	DBPC RCT, single center, 2 arms	Adults	62	Postop: scheduled IV ibuprofen, scheduled oral paracetamol	Rescue opioids	Placebo: saline	Mean ± SD opioid use was 58% lower in the ibuprofen group (26.3 ± 28.7 mg, vs. 62.5 ± 63.8 mg in the placebo group; *p* < 0.0001). The pain score was 43% lower in the ibuprofen group (1.7 ± 2.2, vs. 3.0 ± 2.8 in the placebo group; *p* < 0.0001).	There were two AEs, probably related to IV ibuprofen: a burning sensation at the infusion site, and postoperative hyperkalemia
Various types of abdominal or orthopedic surgery	Zhou et al. 2023 [68]	DBPC RCT, multicenter, 3 arms	18 to 75	345	IV ibuprofen 400 mg, or IV ibuprofen 800 mg or placebo (30 min before end of surgery then every 6 h)	IV morphine (0.5 mg/mL) as PCA	Placebo: not reported	Total morphine consumption was significantly lower in the ibuprofen 400 mg group (11.14 ± 7.14 mg; *p* = 0.0011) and the ibuprofen 800 mg group (11.29 ±6.45 mg; *p* = 0.0014) than in the placebo group (14.51 ± 9.19 mg)	No difference in the incidence of AEs between groups
Various types of urogynecological surgery	Dwarica et al. 2020 [47]	Prospective, RCT, single center, 3 arms	Over 18. Mean (SD) = 55.52 (14.30) in the ibuprofen group	224	IV ibuprofen 800 mg IV every 8 h for 3 doses. IV ketorolac 30 mg IV (15 mg if >65 years of age or <50 kg).	Hydromorphone as PCA, oral paracetamol 650 mg every 6 h	IV ketorolac 30 mg IV (15 mg if >65 years of age or <50 kg) or ibuprofen oral (800 mg)	Levels of pain control and satisfaction were similar in the IV ketorolac and IV ibuprofen groups	Not reported

DB: double-blind; DBPC: double-blind, placebo-controlled; RCT: randomized clinical trial; SB: single-blind; FDC: fixed-dose combination; IV: intravenous; SPID48: sum of the pain-intensity difference over the 48-h period; PCA: patient-controlled analgesia; VAS: visual analog scale; AE: adverse event.

## Data Availability

The data supporting the conclusions of this article are included within the article.
